# Endovascular Treatment of Posttraumatic Pseudoaneurysm of the Common Carotid Artery

**DOI:** 10.1155/2015/427040

**Published:** 2015-03-31

**Authors:** Diego Rojas, Stefan Stefanov, Luis Riera del Moral, Jesús Álvarez, Luis Riera de Cubas

**Affiliations:** Department of Vascular Surgery, La Paz University Hospital, 28046 Madrid, Spain

## Abstract

Carotid artery injuries with pseudoaneurysm are uncommon but associated with central neurologic dysfunction. We present a case of posttraumatic pseudoaneurysm of the right common carotid artery treated by implantation of a covered stent. A 44-year-old woman with multiple injuries after fall from height presents a small dissection flap of the right common carotid artery (RCCA) on the initial computed tomography angiography (CTA). Fifteen days later a 10 mm pseudoaneurysm is observed on control CTA. We decided endovascular treatment. Through right femoral access with a long introducer sheath placed in the innominate artery, we implanted a covered stent Advanta V12 9 × 38 mm in the RCCA. The patient was discharged from the hospital with antiplatelet therapy without any neurological dysfunction and complete exclusion of the pseudoaneurysm. Use of covered stents has emerged as a safe and effective alternative to surgical repair of carotid injuries.

## 1. Introduction

Extracranial carotid artery pseudoaneurysms and aneurysms are extremely rare, altogether accounting for only 0.4–4% of all peripheral artery aneurysms [1, 2]. Various etiologies have been described including traumatic injury, atherosclerotic degeneration, and fibromuscular dysplasia. Because of the risk of rupture as well as the neurological sequelae caused by cerebral atheroembolism, surgical intervention is recommended for carotid artery pseudoaneurysms. Studies have demonstrated the incidence of carotid pseudoaneurysms ranging from 0.02% to 0.4% among all trauma patients [3]. The anatomical location and the coexistence of lesions in other parts of the body make the diagnosis and treatment a challenge in trauma patients. Endovascular techniques with stent-graft exclusion have emerged as an efficient and safe alternative to open surgical resection [4]. We present a case of posttraumatic pseudoaneurysm of the right common carotid artery treated with endovascular stenting.

## 2. Case Presentation

**Figure 1:**
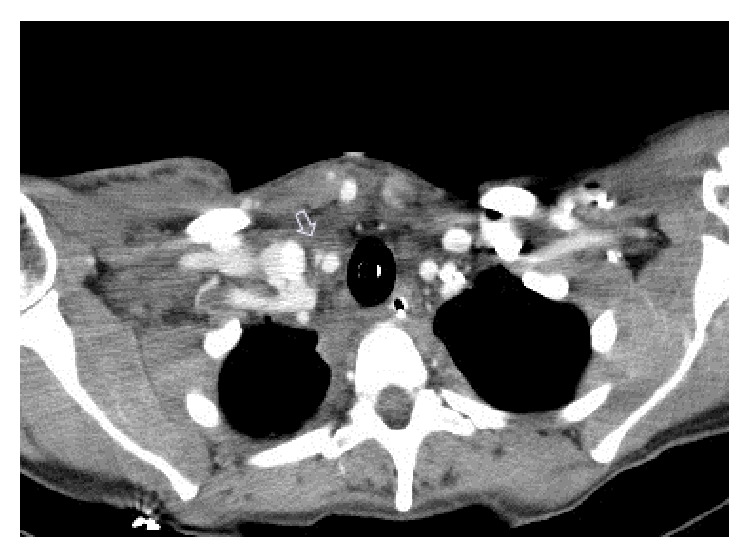
The initial computed tomography (CT) scan revealed a small dissection in the right common carotid artery (white arrow).

**Figure 2:**
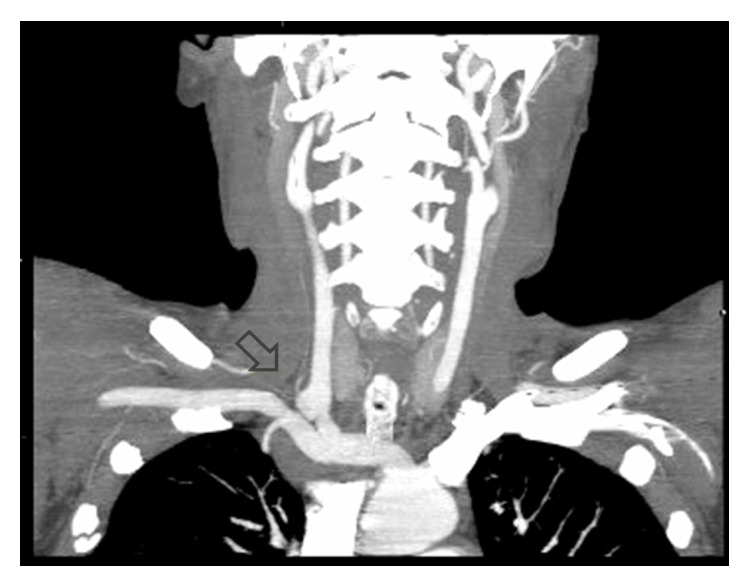
CTA shows a 10 mm pseudoaneurysm in the right common carotid artery (black arrow).

**Figure 3:**
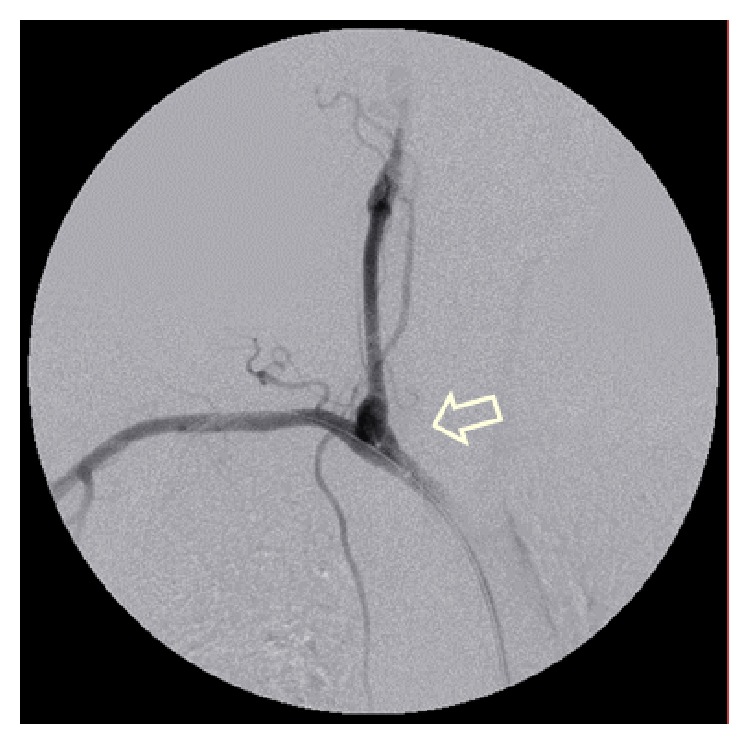
Catheterization of the innominate and subclavian arteries with a 0.035-inch guidewire and a multipurpose catheter. The presence of the pseudoaneurysm was confirmed (white arrow).

**Figure 4:**
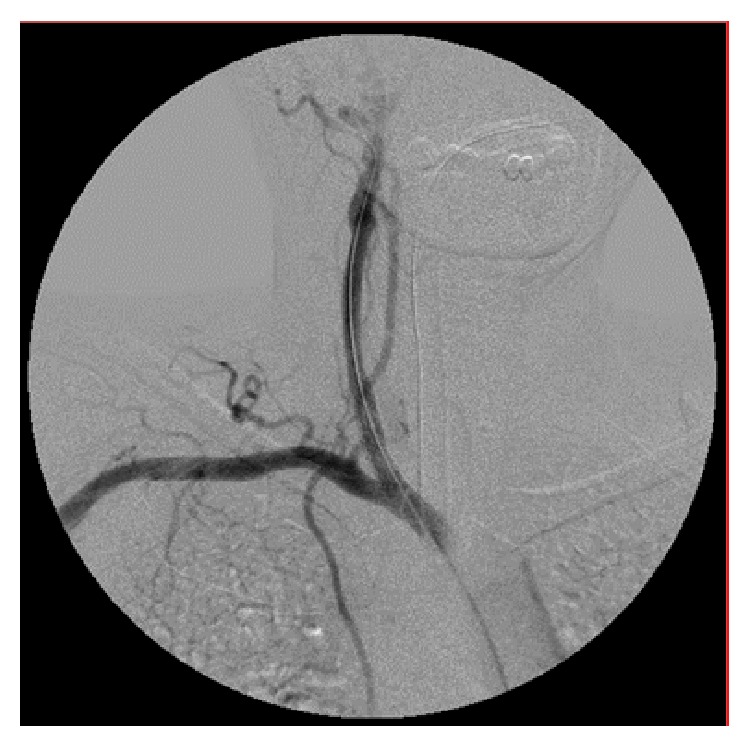
Control angiography showed total exclusion of the pseudoaneurysm. A covered stent was implanted in the right common carotid artery.

A 44-year-old woman was carried to the emergency department in a coma status after fall from height in a suicide attempt. Her only antecedent was depressive syndrome. The initial computed tomography angiography (CTA) scan revealed a small dissection in the right common carotid artery approximately 2 cm from its origin (Figure 1). Fifteen days after the injury a control CTA showed a 10 mm pseudoaneurysm in the same location (Figure 2). Other associated lesions at the moment of arrival were hemorrhagic right brain contusions, pneumothorax with lung contusions, and rib and vertebral fractures. Because of the progression of the lesion and its anatomical location we decided to exclude the pseudoaneurysm with endovascular stenting. Procedure was as follows: through right femoral artery access with a 5Fr introducer sheath and a 0.035-inch guidewire (Terumo Medical Corporation, Japan) an angiography of the aortic arch was performed through a Pigtail catheter. Catheterization of the innominate and subclavian arteries was performed with a 0.035-inch guidewire and a multipurpose catheter that were exchanged for 0.035-inch rigid guidewire (Amplatz, Boston Scientific Corporation, USA) and a 7Fr 90 cm long introducer sheath placed on the distal portion of the innominate artery (Figure 3). The presence of the pseudoaneurysm was confirmed by intraoperative angiography. Using a 0.035-inch rigid guidewire inserted in the external carotid artery to gain support we implanted a covered stent (Advanta V12 9 × 38 mm, Atrium Medical, USA) in the right common carotid artery. Control angiography showed total exclusion of the pseudoaneurysm (Figure 4). No complications occurred during the procedure. The patient was discharged from the hospital with dual antiplatelet therapy 40 days later without any neurological dysfunction or complication related with the intervention. We want to state that the patient provided informed consent for her information and images to be included in this paper and hospital IdiPAZ Clinical Research Ethics Committee provided authorization to submit this case report.

## 3. Discussion

Trauma and prior carotid repair are the most common causes of carotid pseudoaneurysms [5]. Other causes include atherosclerosis, infection, and rare etiologies such as collagen vascular disease, fibromuscular dysplasia, irradiation, and Behçet's disease [6].

Carotid injuries are difficult to evaluate and treat owing to very complex anatomy confined to a relatively narrow anatomic space. Injury in these vessels can be the result of blunt, penetrating, or iatrogenic trauma. Carotid trauma tends to be blunt or stabbing in civilian population. Blunt carotid artery injury (BCI) has been reported with an overall percentage ranging from 0.08% to 0.38% [7]. Blunt carotid injuries have been associated with mortality rates of 20% to 40% and permanent neurologic impairment in 40% to 80% [8, 9]. Penetrating injuries are associated with a mortality rate ranging from 6.6% to 33% [10].

Several types of injuries may result from carotid trauma regardless of mechanism. These injuries include intimal flaps, dissection, pseudoaneurysm, thrombosis, and transaction [10]. The evaluation and management of carotid injury have been controversial and continue to evolve. BCIs typically present with contralateral sensory or motor deficit, decreased mental status, or neurologic deficits not explained by closed head injury [1, 2, 11]. Patients typically have coexisting traumatic brain injuries that may mask signs and symptoms of blunt cerebrovascular injuries [11]. Furthermore, many patients are initially asymptomatic and develop symptoms after a latent period, ranging from 1 hour to several weeks after injury [12]. CTA should be the initial diagnostic step in patients with suspected neck vascular injuries but without hard sings of vascular injury [2]. The most common presentation of carotid pseudoaneurysms is central neurologic dysfunction, regardless of the cause [12, 13]. These events are due to embolization of thrombus from the pseudoaneurysm [11].

The management of traumatic injuries of the carotid artery is influenced by many factors, including the mechanism, anatomical location, type of injury, and associated lesions. The natural history and appropriate management of these injuries remain ill-defined. The cornerstone of treatment for BCI is antithrombotic therapy and anticoagulation is now known to be associated with improved outcome after blunt trauma, but some types of injuries are more likely to fail conservative therapy [2]. Although some small intimal injuries due to blunt mechanism will respond well to conservative approaches including anticoagulation, Panetta et al. have demonstrated that only up to a third of these types of injuries resolve without subsequent complication [14]. Twenty-nine percent of dissections treated with anticoagulation progress to pseudoaneurysm on repeat imaging; pseudoaneurysms themselves classically fail to resolve with anticoagulation alone and constitute continued risk of embolic stroke and rupture if not addressed more aggressively [2].

In the modern era, surgical repair of carotid artery injuries is associated with mortality rates of 0% to 22% and postoperative progression of neurologic deficit of 0% to 21% [10]. Endovascular approaches are increasingly used more frequently in carotid injuries. Stenting has most commonly been used for high extracranial internal carotid lesions as pseudoaneurysms [4, 15]. Compared with surgical treatment of carotid injuries, with an associated mortality rate of up to 22%, carotid stenting appears to be much lower at 0.9% [4, 7, 10, 15]. In addition, stroke rates associated with carotid stenting of trauma, at 3.5%, appear comparable to those after operative repair (0–21%) [10]. The exact timing of stenting is an ill-defined issue in the literature. Some authors suggest delay of carotid stenting, while others recommend delaying carotid stenting until approximately 1 week [15]. Currently no device is specifically designed for this type of injury, and there are no guidelines for the management of carotid artery trauma with endovascular techniques. There is no meta-analysis comparing different endovascular options against each other or against open repair. In a review of DuBose et al., endovascular stent placement was successful in 76.1% of patients with traumatic carotid injuries, with a follow-up period of 2 years and a patency of 79.6% [10]. Martinakis et al. made a systematic review of endovascular management of carotid injury; the treatment of 158 patients between 1997 and 2010 was described [11]. The distribution of carotid injury was BCI (60%), penetrating trauma (29%), and iatrogenic trauma (11%). Pseudoaneurysm was the most frequent lesion (68%) and the most common treatment modality for this type of lesion was placement of self-expanding covered stent in 54% of patients. Other modalities described were use of self-expanding stents (35%), stenting and coiling (20%), and balloon expanding stent (5%) [11]. Seth et al. reported 50 endovascular interventions in 47 patients with internal carotid artery injuries including dissections and pseudoaneurysms. They used stents in 88% of cases (type of stent not specified), coils and stents in 8%, and coils only in 4%. They reported no mortality or neurological dysfunction related to endovascular repair, with a follow-up ranging from 6 months to 7 years [16]. Endovascular repair with covered stents has also been described for the treatment of infected carotid pseudoaneurysms (mycotic pseudoaneurysms) but is limited by the risks associated with introducing prosthetic material into an infected field [17]. Deployment of coils, as an alternative to ligation, in the distal and proximal internal carotid artery was also described, but coils represent a potential cause of persistent infection despite the thrombosis of the pseudoaneurysm [17, 18]. Currently resection of the aneurysm and restoration of arterial supply to the brain through vein graft interpositioning is the preferred procedure along with long-term antibiotic treatment [17].

In this case of posttraumatic pseudoaneurysm we believe that endovascular treatment was the best option. We decided to perform endovascular treatment because of the presence of multiple associated injuries, progression of the lesion, and its anatomical location. We preferred to use a covered balloon expandable stent because it has a precise deployment system. The procedure was carried out without any complication and the patient did not present neurological sequelae. One year later the patient is asymptomatic and the pseudoaneurysm is completely excluded on CT imaging.

In summary, injuries of the carotid artery are uncommon but are serious consequences associated with either blunt or penetrating cervical trauma. They are difficult to evaluate due to associated injuries. Carotid artery stenting has emerged as a safe and effective alternative to surgical repair of carotid injuries. Endovascular treatment of traumatic carotid artery injuries continues to evolve. Further prospective analysis of the role for endovascular treatment of carotid injuries is warranted.
